# Epidemiology, outcomes, and utilization of intensive care unit resources for critically ill COVID-19 patients in Libya: A prospective multi-center cohort study

**DOI:** 10.1371/journal.pone.0251085

**Published:** 2021-04-30

**Authors:** Muhammed Elhadi, Ahmed Alsoufi, Abdurraouf Abusalama, Akram Alkaseek, Saedah Abdeewi, Mohammed Yahya, Alsnosy Mohammed, Mohammed Abdelkabir, Mohammed Huwaysh, Emad Amkhatirah, Kamel Alshorbaji, Samer Khel, Marwa Gamra, Abdulmueti Alhadi, Taha Abubaker, Mohamed Anaiba, Mohammed Elmugassabi, Muhannud Binnawara, Ala Khaled, Ahmed Zaid, Ahmed Msherghi

**Affiliations:** 1 Faculty of Medicine University of Tripoli, Tripoli, Libya; 2 Almwasfat Isolation Hospital, Tripoli, Libya; 3 Gharyan Hospital, Gharyan, Libya; 4 Sebha Medical Center, Sebha, Libya; 5 Almarj Teaching Hospital, Almarj, Libya; 6 Alshahid Attia Alkasah General Hospital, Alkufra, Libya; 7 Misurata Medical Center, Misurata, Libya; 8 Soq Altholatha Isolation Center, Tripoli, Libya; 9 Emeitiga Military Hospital, Tripoli, Libya; 10 Sorman Isolation Center, Sorman, Libya; 11 Faculty of Medicine, University of Zawia, Az Zawiyah, Libya; 12 Benghazi Medical Center, Benghazi, Libya; 13 Tripoli Central Hospital, Tripoli, Libya; Heidelberg University Hospital, GERMANY

## Abstract

**Background:**

The coronavirus disease (COVID-19) pandemic has severely affected African countries, specifically the countries, such as Libya, that are in constant conflict. Clinical and laboratory information, including mortality and associated risk factors in relation to hospital settings and available resources, about critically ill patients with COVID-19 in Africa is not available. This study aimed to determine the mortality and morbidity of COVID-19 patients in intensive care units (ICU) following 60 days after ICU admission, and explore the factors that influence in‐ICU mortality rate.

**Methods:**

This is a multicenter prospective observational study among COVID-19 critical care patients in 11 ICUs in Libya from May 29th to December 30th 2020. Basic demographic data, clinical characteristics, laboratory values, admission Sequential Organ Failure Assessment (SOFA) score, quick SOFA, and clinical management were analyzed.

**Result:**

We included 465 consecutive COVID-19 critically ill patients. The majority (67.1%) of the patients were older than 60 years, with a median (IQR) age of 69 (56.5–75); 240 (51.6%) were male. At 60 days of follow-up, 184 (39.6%) were discharged alive, while 281 (60.4%) died in the intensive care unit. The median (IQR) ICU length of stay was 7 days (4–10) and non-survivors had significantly shorter stay, 6 (3–10) days. The body mass index was 27.9 (24.1–31.6) kg/m2. At admission to the intensive care unit, quick SOFA median (IQR) score was 1 (1–2), whereas total SOFA score was 6 (4–7). In univariate analysis, the following parameters were significantly associated with increased/decreased hazard of mortality: increased age, BMI, white cell count, neutrophils, procalcitonin, cardiac troponin, C-reactive protein, ferritin, fibrinogen, prothrombin, and d-dimer levels were associated with higher risk of mortality. Decreased lymphocytes, and platelet count were associated with higher risk of mortality. Quick SOFA and total SOFA scores increase, emergency intubation, inotrope use, stress myocardiopathy, acute kidney injury, arrythmia, and seizure were associated with higher mortality.

**Conclusion:**

Our study reported the highest mortality rate (60.4%) among critically ill patients with COVID-19 60 days post-ICU admission. Several factors were found to be predictive of mortality, which may help to identify patients at risk of mortality during the ongoing COVID-19 pandemic.

## 1 Introduction

Since the outbreak of severe acute respiratory syndrome coronavirus 2 (SARS-CoV-2) in December 2019, the coronavirus disease (COVID-19) has affected more than 137 million people and has resulted in the death of more than 2.96 million patients by April 14th, 2021 [1]. Symptoms range from mild to severe or life-threatening and may require intensive care unit (ICU) admission. The major cause of morbidity and mortality in COVID-19 is the evolution of acute respiratory distress syndrome (ARDS) and multi-organ dysfunction [2–4].

Several factors, such as increasing age, presence of other comorbidities, and abnormalities in specific laboratory results, have been identified as risk factors for COVID-19 severity [5]. Previous reports have suggested that up to 29% of patients develop ARDS and require hospitalization [2]. An early report suggested that 2–12% of patients will require mechanical ventilation and that mortality can range from 26% to 97% [6]. The rate of COVID-19 patients admitted to ICUs ranged between 7% and 26%, depending on the country. Of 138 hospitalized patients in China, 26.1% were transferred to the ICU [7]. In another study of 1099 patients from 522 hospitals in China, 5% were admitted to the ICU [4]. In Italy, 16% of hospitalized patients and 5–12% of all patients were transferred to the ICU [8, 9]. In the United States, a study of 5700 hospitalized patients in New York found that 20% required ICU admission and mechanical ventilation [10]. Another study in the United States found that 4.9% to 11.5% of 2449 hospitalized patients were admitted to the ICU [11].

Patients requiring ICU admission have been reported to have higher rates of mortality and morbidity during ICU admission. Although rates vary according to geographical and income categories, ICU mortality was 26% among 1591 admitted Italian patients [3]. Another study in China reported that among 344 ICU patients, the mortality was 38.7% within 28 days after ICU admission [12]. Another study in Spain found a mortality rate of 23.2% among 237 patients admitted to the ICU [13]. A European study conducted in France, Belgium, and Switzerland found a 28.1% mortality rate in the ICU in a study of 4224 patients [14]. In a meta-analysis of 24 studies outside of Africa, ICU mortality was 41.6% (34.0–49.7%) [15]. However, the only study from the African region was that of Bruce Biccard et al. [16], which found a 48.2% mortality rate among patients in several African countries. However, there have been no other reports on the survival and mortality rates of African ICU patients. Differences in local settings, as well as differences in predisposing factors in a war-torn country, such as Libya, play a major role in increasing mortality and morbidity.

The COVID-19 pandemic has caused unprecedented challenges for ICUs worldwide. Africa was severely affected by the COVID-19 pandemic due to the lack of available resources and shortage of equipment, along with a lack of properly trained healthcare workers [17, 18]. These major challenges have resulted in the highest ICU mortality in Africa compared to that in the rest of the world, and Bruce et al. [16] reported a 54.7% mortality rate for African patients after 30 days of ICU admission. This high ratio reflects the severe shortage of healthcare infrastructure and the requirement for facilities in order to fight COVID-19.

In Libya, the first laboratory-confirmed case of COVID-19 was detected on March 24, 2020 [19]. Subsequently, the pandemic increased steeply to approximately 170,000 cases and 2,830 deaths by April 14th, 2021. Libya was not prepared for the COVID-19 pandemic. It had a severe shortage of ICU beds and mechanical ventilators [20]. In addition, 56.7% of healthcare workers reported a shortage of personal protective equipment (PPE), and 70% reported purchasing PPE themselves [20]. In this context, assessing mortality and complications, along with ICU needs, is crucial to provide an overview of the current COVID-19 situation in some developing African countries where conducting research is very challenging.

Our main objective was to determine the mortality and morbidity of COVID-19 patients 60 days after ICU admission, and to examine individual risk factors associated with ICU mortality.

## 2 Materials and methods

### 2.1 Study and selection of patients

This is a prospective cohort study of COVID-19 patients consecutively admitted to ICUs in 11 hospitals and isolation centers that managed COVID-19 patients in Libya from the 29th of May,2020 to the 30th of December 2020. All hospitals are public hospitals funded by the government, as private hospitals do not admit COVID-19 patients. Patients recruited in the study were followed up for 60 days after ICU admission or until discharge. The hospitals that participated in the study were originally mixed surgical-medical specialized COVID-19 centers with daily multidisciplinary rounds and specific protocols for patient care during the pandemic. The study included only patients whose COVID-19 infection was confirmed by real-time PCR (RT-PCR) assay for SARS-CoV-2 testing of a nasopharyngeal sample. Inclusion criteria were as follows: adults aged > 18 years admitted to the ICU. Exclusion criteria were negative RT-PCR for COVID-19 or patients transferred to wards or isolation departments. The primary outcome was 60-day mortality after ICU admission.

**Fig 1:**
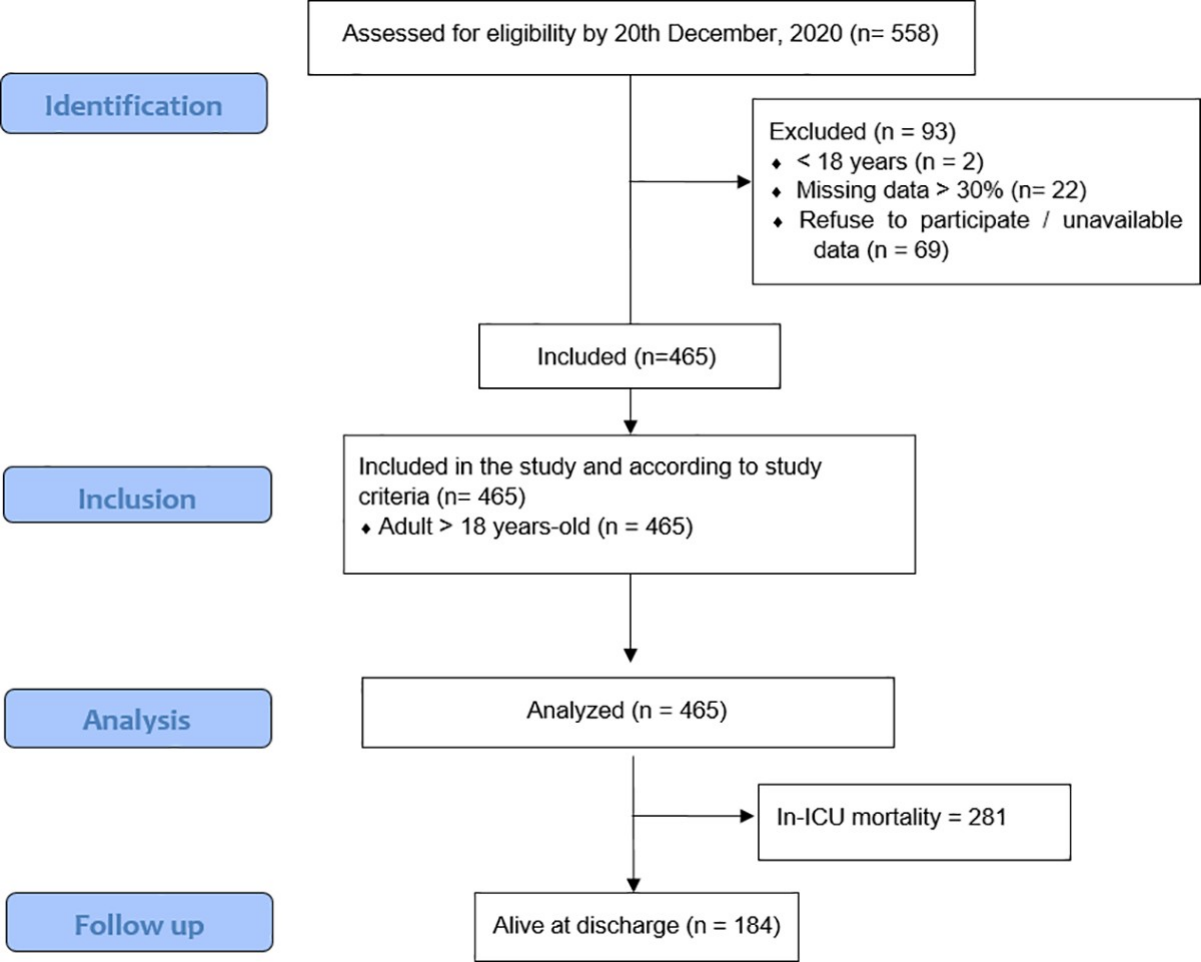
STROBE flow chart. STROBE, Strengthening the Reporting of Observational Studies in Epidemiology.

The COVID-19 patient data recorded included basic demographic data, essential medical history, pre-admission medication history, comorbidities, antibiotic and antifungal history, radiological results, applied treatment, organ support indications, intubation history and details, thromboembolic complications and treatment, temperature, basic laboratory results, admission sequential organ failure assessment (SOFA) score, ICU length of stay in days, and status at hospital discharge or 60 days after critical care admission. SOFA score is an objective score, which allows for measurement in six organ systems (respiratory, coagulatory, hepatic, cardiovascular, renal and neurological), with the score measuring individual or multiple organ dysfunction [21, 22]. Each organ has a score range from 0 to 4. SOFA scoring system is used to predict the clinical outcomes of patients admitted to intensive care units [21, 23]. The study adhered to the Strengthening the Reporting of Observational Studies in Epidemiology statement [24], and the recruitment strategy is summarized in a flow chart in **Fig 1**.

In Libya, there is no provision for extracorporeal membrane oxygenation (ECMO); in addition, there are no guidelines or policies for withdrawal of life support or to make decisions to limit therapy.

### 2.2 Statistical analysis

The Shapiro–Wilk normality test was used to determine the non-normal distribution of continuous variables. Median and interquartile ranges were used for continuous variables, while categorical variables were presented as frequencies and percentages. For continuous variables, the Mann–Whitney or Kruskal–Wallis H tests were used to determine statistically significant differences between two or more groups of an independent variable and a continuous variable. The chi-square test was used for categorical variables for association and comparison between survivor and non-survivor groups. Phi (φ) and Cramer’s V were used to determine the impact of association in the chi-square test for two or more variables, respectively. Cox regression was performed to calculate hazard ratios (HRs) and 95% confidence intervals for both the mortality and ICU admission outcome variables, where the binary outcome was (survival/death), and the length of stay in the ICU was used as a measurement of time. Univariate and multivariate Cox regression analyses were performed. Kaplan–Meier survival analysis (Kaplan & Meier, 1958) was conducted to compare the study variables and the impact on ICU mortality. A log-rank test was performed to determine the significance of differences in the survival distribution for the study variables and intervention suggested. All tests were two-tailed and considered significant when p < 0.05. Statistical analysis was performed using IBM SPSS Statistics for Windows (IBM SPSS Statistics for Windows, Version 25.0, IBM Corp., Armonk, NY, USA).

### 2.3 Ethical approval

Ethical approval for this study was obtained from the Bioethics Committee at the Biotechnology Research Center in Libya (Reference number: BEC-BTRC-13-2020). Informed consent was not required due to the observational nature of the study and the pandemic situation.

## 3 Results

### 3.1 Patient baseline characteristics

The study included 465 critically ill COVID-19 patients in this study. The study excluded 69 patients, including patients who refused to participate in the study, two who were < 18 years of age, and 22 patients for whom > 30% of data were not available (study enrollment flowchart is shown in Fig 1). At 60 days of follow-up, 184 (39.6%) patients were discharged alive from the hospital, whereas 281 (60.4%) died in the ICU.

The majority of the patients were older than 60 years, with a median (IQR) age of 69 (56.5–75) years; 240 (51.6%) were male and 225 (48.4%) were female. The body mass index of the participants was 27.9 (24.1–31.6). Of the patients included, only eight (1.7%) were healthcare workers and five (1.1%) were pregnant women. A total of 264 (56.8%) patients had diabetes, and 259 (55.7%) had arterial hypertension. Among the study participants, 225 (48.4%) were transferred from the emergency department to the ICU, 121 (26%) were transferred from the in-hospital ward, and 119 (25.6%) were transferred from another hospital or healthcare facility. None of the patients included in the study had a history of human immunodeficiency virus (HIV) infection. The characteristics of the study participants are listed in **Table 1,** with a comparison between surviving and non-surviving patients. Median laboratory values were significantly different between survivors and non-survivors; white blood cells, neutrophils, procalcitonin, cardiac troponin, ferritin, fibrinogen, prothrombin, and D-dimer levels were higher in non-survivors than in survivors, while lymphocyte counts and platelet counts were significantly lower in non-survivors than in survivors.

**Table 1:** Basic and laboratory characteristics of COVID-19 patients in the intensive care unit during admission.

Variables	All patients (%) n = 465	Survivor (%) n = 184	Non-survivor (%) n = 281	χ2	P-value
	n (%) or Median (Q1–Q3)	n (%) or Median (Q1–Q3)	n (%) or Median (Q1–Q3)		
**General characteristics**					
** Age, years**	69 (56.5–75)	65 (55–72)	70 (60–78.5)		<0.001**
** Age range (years)**				0.23	<0.001**
** **≤ 60	153 (32.9%)	81 (44%)	72 (25.6%)		
** **60–70	119 (25.6%)	50 (27.2%)	69 (24.6%)		
** **> 70	193 (41.5%)	53 (28.8%)	140 (49.8%)		
** Gender**				0.07	0.15
** **Female	225 (48.4%)	97 (52.7%)	128 (45.6%)		
** **Male	240 (51.6%)	87 (47.3%)	153 (54.4%)		
** Body mass index (kg/m2)**	27.9 (24.1–31.6)	25 (21.9–28.8)	29.8 (25.8–33.6)		<0.001**
** Temperature in °C**	38 (37.4–38.5)	38 (37.5–38.7)	37.9 (37.3–38.5)		0.12
** Healthcare workers**	8 (1.7%)	5 (2.7%)	3 (1.1%)	0.18	0.27
** Pregnant**	5 (1.1%)	2 (1.1%)	3 (1.1%)	0.98	1
**Comorbidities (n, %)**					
** **Diabetes	264 (56.8%)	97 (52.7%)	167 (59.4%)	0.06	0.18
** **Chronic Cardiac Disease (Not HTN)	97 (20.9%)	40 (21.7%)	57 (20.3%)	0.02	0.72
** **Hypertension	259 (55.7%)	102 (55.4%)	157 (55.9%)	0.004	1
** **Chronic Pulmonary Disease	36 (7.7%)	16 (8.7%)	20 (7.1%)	0.03	0.59
** **Asthma	51 (11%)	25 (13.6%)	26 (9.3%)	0.06	0.17
** **Chronic Kidney Disease	45 (9.7%)	14 (7.6%)	31 (11%)	0.05	0.26
** **Chronic Liver Disease	5 (1.1%)	1 (0.5%)	4 (1.4%)	0.04	0.65
** **Neurological Disorder	28 (6%)	12 (6.5%)	16 (5.7%)	0.02	0.69
** **Malignant Neoplasm	18 (3.9%)	3 (1.6%)	15 (5.3%)	0.09	0.05
** **Immunosuppression	4 (0.9%)	0 (0%)	4 (1.4%)	0.07	0.15
**History of surgery within 2 weeks**	9 (1.9%)	3 (1.6%)	6 (2.1%)	0.02	1
**Total number of comorbidities (n, %)**				0.038	0.87
** **No comorbidities	90 (19.4%)	36 (19.6%)	54 (19.2%)		
** **One comorbidity	113 (24.3%)	46 (25%)	67 (23.8%)		
** **Two comorbidities	131 (28.2%))	54 (29.3%)	77 (27.4%)		
** **Three or more comorbidities	131 (28.2%	48 (26.1%)	83 (29.5%)		
**Drug history taking in 14-days prior to admission (n, %)**					
** **Angiotensin Converting Enzyme Inhibitor	105 (22.6%)	50 (27.2%)	55 (19.6%)	0.09	0.06
** **Angiotensin II Receptor Blocker	57 (12.3%)	12 (6.5%)	45 (16%)	0.14	0.002*
** **Anticoagulation	39 (8.4%)	13 (7.1%)	26 (9.3%)	0.04	0.4
** **Antiplatelet	113 (24.3%)	51 (27.1%)	62 (22.1%)	0.06	0.16
**Type of respiratory support at admission (n, %)**				0.17	0.006*
** **Did not receive respiratory support	203 (43.7%)	74 (40.2%)	129 (45.9%)		
** **Standard Oxygen Mask	222 (47.4%)	103 (56%)	119 (42.3%)		
** **Non-invasive ventilation (NIV)	2 (0.4%)	0 (0%)	2 (0.7%)		
** **High-flow nasal oxygen ** **(HFNO)	20 (4.3%)	5 (2.7%)	15 (5.3%)		
** **Continues positive airway pressure therapy (CPAP)	18 (3.9%)	2 (1.1%)	16 (5.7%)		
**Bacterial pulmonary co-infection at admission**	195 (41.9%)	68 (37%)	127 (45.2%)	0.08	0.084
**Laboratory findings during admission**					
** **Routine blood test					
** **White blood cells, ✕ 10^9^/L	8.1 (5.4–14.1)	4.98 (3.5–6.9)	13 (8.6–16.4)		<0.001**
** **Neutrophils, ✕ 10^9^/L	6.6 (4.8–9.71)	4.7 (2.8–6.3)	8.4 (6.4–10.6)		<0.001**
** **Lymphocytes, ✕ 10^9^/L	0.7 (0.5–0.9)	1.03 (0.7–1.3)	0.6 (0.4–0.8)		<0.001**
** **Platelets, ✕ 10^9^/L	69.3 (112–213)	203.8 (165–260)	137.2 (75.5–185.7)		<0.001**
** **Other laboratory values					
** **Procalcitonin (PCT), ng/ml	0.28 (0.07–0.59)	0.06 (0.03–0.08)	0.5 (0.3–0.7)		<0.001**
** **High-sensitivity cardiac troponin I, pg/ml	36.9 (5.9–120.4)	4.5 (2.3–7.3)	102.6 (51.8–157.9)		<0.001**
** **C-reactive protein (CRP), mg/L	66.1 (39.7–115.6)	39.8 (21.8–59.03)	103.2 (60.92–139.3)		<0.001**
** **Ferritin (μg/L)	1072.76 (806.2–2129.3)	810.8 (354.7–810)	1804.7 (1042.8–2540.6)		<0.001**
** **Fibrinogen (mg/dl)	491.4 (383.6–615.1)	438.2 (319.4–562.4)	535.8 (422.4–658.8)		<0.001**
** **Prothrombin time (s)	14.5 (12.1–16.5)	13.5 (11.9–15.6)	15.3 (12.7–17.5)		0.04*
** **D-dimer (ng/ml)	6.35 (1.18–21.33)	1.03 (0.59–1.47)	18.1 (9.6–27.1)		<0.001**

### 3.2 Clinical status at admission

At admission to the ICU, the quick SOFA median (IQR) score was 1 (1–2), and the total SOFA score was 6 (range, 4–7). The means (SD) of quick SOFA and SOFA were 1.48 (0.84) and 6.04 (2.53), with a range from 0 to 3 and 1 to 19, respectively. In the non-survivor group (281/465), the quick SOFA and SOFA scores were 1.62 (0.8) and 6.85 (2.63), whereas in the survivor group (184/465) were 1.27 (0.79) and 4.81 (1.75), respectively. In the non-survivor group, the quick SOFA and total SOFA scores ranged from 0 to 3 and 1 to 19, whereas in the survivor group, they ranged from 0 to 3 and 1 to 9, respectively.

When analyzing the composition of the quick SOFA score, 412 (88.6%) patients presented with a respiratory rate ≥ 22, 157 (33.8%) presented with a Glasgow Coma Scale score < 15, and 120 (25.8%) presented with a systolic blood pressure ≤ 100. Regarding the total SOFA score categories, 427 (91.8%) patients presented with a SOFA score of ≤ 9, which predicts a risk of mortality of less than 33.3%, whereas 28 (6%) scored between 10 and 12, which predicts an approximately 50% risk of mortality, and 10 (2.2%) presented with a SOFA score > 12, which predicts a mortality rate of 95.2%. However, none of the survivors had a SOFA score of > 9. The study found a statistically significant association between quick and full SOFA scores and mortality in patients. **Table 2** provides an overview of the SOFA score and quick SOFA score at admission.

**Table 2:** Sequential Organ Failure Assessment (SOFA) and quick SOFA (qSOFA) score at admission.

Variables	All patients (%) n = 465	Survivor (%) n = 184	Non-survivor (%) n = 281	χ2	P-value
	n (%) or Median (Q1–Q3)	n (%) or Median (Q1–Q3)	n (%) or Median (Q1–Q3)		
**Quick SOFA Score**					
Glasgow Coma Scale <15				0.17	<0.001**
Yes	157 (33.8%)	44 (23.9%)	113 (40.2%)		
No	308 (66.2%)	140 (76.1%)	168 (59.8%)		
Respiratory rate ≥22				0.07	0.133
Yes	412 (88.6%)	158 (85.9%)	254 (90.4%)		
No	53 (11.4%)	26 (14.1%)	27 (9.6%)		
Systolic BP ≤100				0.17	<0.001**
Yes	120 (25.8%)	31 (16.8%)	89 (31.7%)		
No	345 (74.2%)	153 (83.2%)	192 (68.3%)		
**Total qSOFA score (0–3)**	1 (1–2)	1 (1–2)	2 (1–2)		<0.001**
**Score category**				0.24	<0.001**
0	46 (9.9%)	23 (12.5%)	23 (8.2%)		
1	209 (44.9%)	105 (57.1%)	104 (37%)		
2	151 (32.5%)	40 (21.7%)	111 (39.5%)		
3	59 (12.7%)	16 (8.7%)	43 (15.3%)		
**Risk group interpretation**				0.24	<0.001**
0–1	255 (54.8%)	128 (69.6%)	127 (45.2%)		
2–3	210 (45.2%)	56 (30.4%)	154 (54.8%)		
**SOFA Score**					
PaO2/FiO2*, mmHg				0.23	<0.001**
≥400	21 (4.5%)	9 (4.9%)	12 (4.3%)		
300–399	137 (29.5%)	67 (36.4%)	70 (24.9%)		
200–299 OR ≤199 and NOT mechanically ventilated	102 (21.9%)	48 (26.1%)	54 (19.2%)		
100–199 and mechanically ventilated	79 (17%)	33 (17.9%)	46 (16.4%)		
<100 and mechanically ventilated	126 (27.1%)	27 (14.7%)	99 (35.2%)		
**Bilirubin, mg/dL (μmol/L)**				0.17	0.009*
<1.2 (<20)	329 (70.8%)	143 (77.7%)	186 (66.2%)		
1.2–1.9 (20–32)	114 (24.5%)	39 (21.2%)	75 (26.7%)		
2.0–5.9 (33–101)	18 (3.9%)	1 (0.5%)	17 (6%)		
6.0–11.9 (102–204)	2 (0.4%)	1 (0.5%)	1 (0.4%)		
≥12.0 (>204)	2 (0.4%)	0 (0%)	2 (0.7%)		
**Mean arterial pressure OR administration of vasopressor**				0.18	0.004*
No hypotension	309 (66.5%)	134 (72.8%)	175 (62.3%)		
MAP <70 mmHg	131 (28.2%)	49 (26.6%)	82 (29.2%)		
Dopamine ≤5 or Dobutamine (any dose)	18 (3.9%)	1 (0.5%)	17 (6%)		
Dopamine >5, Epinephrine ≤0.1, or norepinephrine ≤0.1	6 (1.3%)	0 (0%)	6 (2.1%)		
Dopamine >15, Epinephrine >0.1, or norepinephrine >0.1	1 (0.2%)	0 (0%)	1 (0.4%)		
**Creatinine, mg/dL (μmol/L) (or urine output)**				0.18	0.003*
<1.2 (<110)	210 (45.2%)	101 (54.9%)	109 (38.8%)		
1.2–1.9 (110–170)	181 (38.9%)	63 (34.2%)	118 (42%)		
2.0–3.4 (171–299)	49 (10.5%)	13 (7.1%)	36 (12.8%)		
3.5–4.9 (300–440) or UOP <500 mL/day)	11 (2.4%)	1 (0.5%)	10 (3.6%)		
≥5.0 (>440) or UOP <200 mL/day	14 (3%)	6 (3.3%)	8 (2.8%)		
**Platelets, ×10**^**3**^**/μL**				0.06	0.597
≥150	311 (66.9%)	125 (67.9%)	186 (66.2%)		
100–149	120 (25.8%)	49 (26.6%)	71 (25.3%)		
50–99	26 (5.6%)	7 (3.8%)	19 (6.8%)		
20–49	8 (1.7%)	3 (1.6%)	5 (1.8%)		
<20	0 (0%)	0 (0%)	0 (0%)		
**Glasgow Coma Scale**				0.43	<0.001**
15	1 (0.2%)	1 (0.5%)	0 (0%)		
13–14	248 (53.3%)	145 (78.8%)	103 (36.7%)		
10–12	110 (23.7%)	27 (14.7%)	83 (29.5%)		
6–9	81 (17.4%)	11 (6%)	70 (24.9%)		
<6	25 (5.4%)	0 (0%)	25 (8.9%)		
**Total SOFA score (0–24)**	6 (4–7)	5 (3–6)	7 (5–8)		<0.001**
**Score category**				0.42	<0.001**
1	5 (1.1%)	4 (2.2%)	1 (0.4%)		
2	19 (4.1%)	12 (6.5%)	7 (2.5%)		
3	48 (10.3%)	32 (17.4%)	16 (5.7%)		
4	51 (11%)	28 (15.2%)	23 (8.2%)		
5	76 (16.3%)	43 (23.4%)	33 (11.7%)		
6	94 (20.2%)	38 (20.7%)	56 (19.9%)		
7	66 (14.2%)	15 (8.2%)	51 (18.1%)		
8	41 (8.8%)	7 (3.8%)	34 (12.1%)		
9	27 (5.8%)	5 (2.7%)	22 (7.8%)		
10	14 (3%)	0 (0%)	14 (5%)		
11	9 (1.9%)	0 (0%)	9 (3.2%)		
12	5 (1.1%)	0 (0%)	5 (1.8%)		
13	5 (1.1%)	0 (0%)	5 (1.8%)		
14	2 (0.4%)	0 (0%)	2 (0.7%)		
15	1 (0.2%)	0 (0%)	1 (0.4%)		
16	1 (0.2%)	0 (0%)	1 (0.4%)		
>16	1 (0.2%)	0 (0%)	1 (0.04%)	0.24	<0.001**
**Risk group interpretation**					
≤ 9	427 (91.8%)	184 (100%)	243 (86.5%)		
10–12	28 (6%)	0 (0%)	28 (10%)		
> 12	10 (2.2%)	0 (0%)	10 (3.6%)		

Quick SOFA score, 0–1 not high risk for mortality, 2–3 high risk for mortality.

SOFA score of less than or equal to 9 (it predicts less than 33.33% mortality), score between 10 and 12 (predicts approximately 50% mortality), and a score of more than 12 (it predicts approximately 95.2% mortality).

### 3.3 Intensive care management

**Fig 2:**
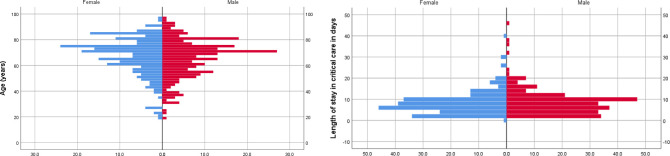
(A) Distribution of mortality of critically ill patients with COVID-19 stratified according to age and sex. (B) Distribution of length of stay in critical care, in days, for COVID-19 patients stratified according to age and mortality.

An overview of the detailed management of ICUs is presented in **Table 3**. The median (IQR) ICU length of stay was 7 days (range, 4–10 days), with a significantly shorter stay among non-survivors. At the end of the study, 460 (98.9%) patients were either discharged or died at 30 days. Only five (1.1%) patients had a hospital stay of more than 30 days; two of them died at 32 and 38 days, and three were discharged at 35, 39, and 45 days of ICU admission, as shown in **Fig 2A**, which illustrates the distribution of mortality according to age, sex, and length of ICU stay, and **Fig 2B**, which shows mortality.

**Table 3:** Admission management and characteristics of COVID-19 patients in the intensive care unit.

Variables	All patients (%) n = 465	Survivor (%) n = 184	Non-survivor (%) n = 281	χ2	P-value
	n (%) or Median (Q1–Q3)	n (%) or Median (Q1–Q3)	n (%) or Median (Q1–Q3)		
**ICU length of stay**	7 (4–10)	8 (5–10)	6 (3–10)		<0.001**
**Airway Management Received During ICU Admission**					
Oxygen Mask	440 (94.6%)	177 (96.2%)	263 (93.6%)	0.05	0.22
Continues positive airway pressure therapy (CPAP)	226 (48.6%)	75 (40.8%)	151 (53.7%)	0.12	0.006
High-flow nasal oxygen (HFNO)	81 (17.4%)	32 (17.4%)	49 (17.4%)	0.001	1
Prone position with mechanical ventilation	54 (11.6%)	6 (3.3%)	48 (17.1%)	0.21	<0.001**
**Type of intubation**				0.57	<0.001**
Elective	38 (8.2%)	4 (2.2%)	34 (12.1%)		
Emergency	173 (37.2%)	14 (7.6%)	159 (56.6%)		
No intubation	254 (54.6%)	166 (90.2%)	88 (31.3%)		
**Developed infection at any point during ICU admission**	154 (33.1%)	40 (21.7%)	114 (40.6%)	0.19	<0.001**
**Course and type of infection developed**				0.25	<0.001**
None	311 (66.9%)	143 (77.7%)	168 (59.8%)		
Sepsis	120 (25.8%)	41 (22.3%)	79 (28.1%)		
Septic shock	34 (7.3%)	0 (0%)	34 (12.1%)		
**ICU management**					
**Inotropes / vasopressor**	122 (26.2%)	10 (5.4%)	112 (39.9%)	0.38	<0.001**
**DVT prophylaxis during first 12 hours**				0.23	<0.001**
None	88 (18.9%)	24 (13%)	64 (22.8%)		
Unfractionated heparin	118 (25.4%)	32 (17.4%)	86 (30.6%)		
Fraxiparine	162 (34.8%)	81 (44%)	81 (28.8%)		
Enoxaparin	55 (11.8%)	28 (15.2%)	27 (9.6%)		
Aspirin	40 (8.6%)	18 (9.8%)	22 (7.8%)		
Other	2 (0.4%)	1 (0.5%)	1 (0.4%)		
**Indications for anticoagulation**				0.21	0.002*
None	80 (17.2%)	25 (13.6%)	55 (19.6%)		
Prophylaxis	326 (70.1%)	145 (78.8%)	181 (64.4%)		
Myocardial Infarction	17 (3.7%)	7 (3.8%)	10 (3.6%)		
Limb ischemia	6 (1.3%)	3 (1.6%)	3 (1.1%)		
Deep venous thrombosis	6 (1.3%)	2 (1.1%)	4 (1.4%)		
Pulmonary embolism	30 (6.5%)	2 (1.1%)	28 (10%)		
**Therapies**					
Steroid	417 (89.7%)	168 (91.3%)	249 (88.6%)	0.04	0.43
Blood transfusion	28 (6%)	15 (8.2%)	13 (4.6%)	0.07	0.11
Convalescent plasma	23 (4.9%)	7 (3.8%)	16 (5.7%)	0.04	0.39
Remdesivir	21 (4.5%)	7 (3.8%)	14 (5%)	0.03	0.65
Tocilizumab	2 (0.4%)	0 (0%)	2 (0.7%)	0.05	0.52
Vitamin C				0.07	0.33
None	188 (40.4%)	82 (44.6%)	106 (37.7%)		
Low dose	79 (17%)	28 (15.2%)	51 (18.1%)		
High dose	198 (42.6%)	74 (40.2%)	124 (44.1%)		
Vitamin D	15 (3.2%)	8 (4.3%)	7 (2.5%)	0.29	0.29
Famotidine	60 (12.9%)	26 (14.1%)	34 (12.1%)	0.03	0.52
**Antimicrobial and antibiotics**					
Antifungal	30 (6.5%)	12 (6.5%)	18 (6.4%)	0.002	1
Antibiotic	433 (93.1%)	181 (98.4%)	252 (89.7%)	0.16	<0.001**
Aminoglycosides	18 (3.9%)	4 (2.2%)	14 (5%)	0.07	0.15
Glycopeptide	52 (11.2%)	14 (7.6%)	38 (13.5%)	0.09	0.05
Cephalosporin	283 (60.9%)	128 (69.6%)	155 (55.2%)	0.14	0.002*
Quinolones	130 (28%)	33 (17.9%)	97 (34.5%)	0.18	<0.001*
Carbapenem	124 (26.7%)	45 (24.5%)	79 (28.1%)	0.04	0.39
Macrolide	221 (47.5%)	111 (60.3%)	110 (39.1%)	0.21	<0.001**
Penicillin	75 (16.1%)	19 (10.3%)	56 (19.9%)	0.13	0.006*
Other antibiotics	4 (0.9%)	1 (0.5%)	3 (1.1%)	0.03	1
**Intensive Care Complications**				0.19	0.01*
Respiratory Complications	435 (93.5%)	171 (92.9%)	264 (94%)		
Myocardial damage	6 (1.3%)	5 (2.7%)	1 (0.4%)		
Rhabdomyolysis	1 (0.2%)	0 (0%)	1 (0.4%)		
Acute kidney injury (AKI)	10 (2.2%)	1 (0.5%)	9 (3.2%)		
Fulminant liver failure	2 (0.4%)	0 (0%)	2 (0.7%)		
Diabetic ketoacidosis (DKA)	4 (0.9%)	1 (0.5%)	3 (1.1%)		
Cerebrovascular accident (CVA)	4 (0.9%)	3 (1.6%)	1 (0.4%)		
Other	3 (0.6%)	3 (1.6%)	0 (0%)		
**Thromboembolic events**					
**Present at arrival**				0.13	0.06
DVT	2 (0.4%)	0 (0%)	2 (0.7%)		
Pulmonary embolism	23 (4.9%)	4 (2.2%)	19 (6.8%)		
Not documented	416 (89.5%)	168 (91.3%)	248 (88.3%)		
Other embolic events	24 (5.2%)	12 (6.5%)	12 (4.3%)		
**During admission**				0.17	0.02*
None	397 (85.4%)	167 (90.8%)	230 (81.9%)		
Stroke and cerebrovascular events	9 (1.9%)	2 (1.1%)	7 (2.5%)		
Myocardial infarction	16 (3.4%)	8 (4.3%)	8 (2.8%)		
Deep venous thrombosis (DVT)	8 (1.7%)	2 (1.1%)	6 (2.1%)		
Pulmonary embolism (PE)	32 (6.9%)	4 (2.2%)	28 (10%)		
DVT + PE	3 (0.6%)	1 (0.5%)	2 (0.7%)		
**Major complications events**					
Life-threatening hemorrhage	11 (2.4%)	2 (1.1%)	9 (3.2%)	0.07	0.21
Stress myocardiopathy	44 (9.5%)	8 (4.3%)	36 (12.8%)	0.14	0.002*
Acute kidney injury	127 (27.3%)	23 (12.5%)	104 (37%)	0.27	<0.001**
Cardiac arrythmia	88 (18.9%)	14 (7.6%)	74 (26.3%)	0.23	<0.001**
Myocarditis	34 (7.3%)	7 (3.8%)	27 (9.6%)	0.11	0.018*
Pericardial effusion	29 (6.2%)	8 (4.3%)	21 (7.5%)	0.06	0.24
Pneumothorax	38 (8.2%)	14 (7.6%)	24 (8.5%)	0.02	0.86
Atelectasis	93 (20%)	28 (15.2%)	65 (23.1%)	0.10	0.037*
Prolonged delirium	75 (16.1%)	15 (8.2%)	60 (21.4%)	0.17	<0.001**
Seizure	42 (9%)	6 (3.3%)	36 (12.8%)	0.15	0.002*
**Pressure ulcer**					
Facial prone ulcer	22 (4.7%)	7 (3.8%)	15 (5.3%)	0.05	0.57
Decubitus ulcer	57 (12.3%)	10 (5.4%)	47 (16.7%)	0.17	0.001*
**Intubation and extubation events**					
Unplanned extubation	18 (3.9%)	4 (2.2%)	14 (5%)	0.07	0.27
Endotracheal tube obstruction	58 (12.5%)	6 (3.3%)	52 (18.5%)	0.23	<0.001**

A total of 154 (33.1%) patients developed infection during their ICU stay. Inotrope use, prone position with mechanical ventilation, thromboembolic events during admission, stress myocardiopathy, acute kidney injury, arrhythmia, myocarditis, atelectasis, prolonged delirium, seizures, decubitus ulcers, and endotracheal tube obstruction were significantly higher in the non-survivor group than in the survivor group. Antibiotics were administered to 433 (93.1%) patients; antibiotics, especially quinolones, macrolides, and penicillin, were significantly associated with the survivor group.

Steroids were not associated with any group, as both received steroids at similar rates. Other therapies, such as convalescent plasma, blood transfusion, Remdesivir, Tocilizumab, Famotidine, and high or low doses of vitamin C or vitamin D, did not show significant association with either group due to the low frequency of use of these drugs. Respiratory complications were the most frequent, accounting for 435 cases (93.5%) of intensive care complications. A total of 127 cases of acute kidney injury (27.3%), 93 cases of atelectasis (20%), and 88 cases of cardiac arrhythmia (18.9%) were also major events during ICU stay. Thromboembolic events were higher during admission than during pre-admission. An overview of the detailed complications and management of ICU patients is presented in **Table 3**.

### 3.4 Factors associated with survival

Patients were categorized into survivors (184/465) and non-survivors (281/465) at the end of the study follow-up, or at discharge or death. Univariate and multivariate Cox proportional regression hazard analyses were performed on the study variables, as detailed in **Table 4**. Multivariate analysis showed that only the following variables were significantly associated with mortality: lower lymphocyte count (HR = 0.35 [0.18, 0.68]), higher procalcitonin (HR = 1.89 [1.03, 3.46]), cardiac troponin (HR = 1.004 [1.002, 1.006]), C-reactive protein (HR = 1.004 [1.001, 1.007]), D-dimer (HR = 1.02 [1.001, 1.03]), total SOFA score (HR = 1.16 [1.07, 1.25]), emergency intubation (HR = 1.97 [1.37, 2.85]), and stress cardiomyopathy (HR = 2.31 [1.27, 4.16]). In univariate analysis, the following were significantly associated with increase/decrease hazard of mortality: increased age (HR = 1.0 [1, 1.02]), BMI (HR = 1.08 [10.6, 1.11]), white cell count (HR = 1.12 [1.09, 1.14]), neutrophil (HR = 1.07 [1.05–1.1]), procalcitonin (HR = 7.83 [5.25, 11.65]), cardiac troponin (HR = 1.009 [1.007, 1.011]), C-reactive protein (HR = 1.011 [1.009–1.014]), ferritin (HR = 1.00 [1.00, 1.001]), fibrinogen (HR = 1.002 [1.001–1.003]), prothrombin (HR = 1.09 [1.04, 1.14]), and D-dimer (HR = 1.05 [1.04, 1.06]). Decreased lymphocyte (HR = 0.14 [0.08, 0.21]) and platelet count (HR = 0.993 [0.991, 0.995]) were both associated with increased mortality. However, increased antibiotic use reduced mortality (HR = 0.56 [0.38, 0.83]). Both increased quick SOFA score (HR = 1.05 [1.04, 1.06]), and increased total SOFA score (HR = 1.12 [1.08, 1.26]) increased the risk of mortality.

**Table 4:** Association of patient characteristics with 60-day post-ICU admission mortality using ICU length of stay as time variable (univariate/multivariate cox regression models).

Parameters	All patients (%) n = 465 n (%) or Median (Q1–Q3)	Univariate analysis		Multivariate analysis	
		Hazzard ratio (95% confidence intervals)	p-value	Hazzard ratio (95% confidence intervals)	p-value
**Age in years**	69 (56.5–75)	1.0 (1–1.02)	0.038*	0.99 (0.97–1.02)	0.58
**Age range (years)**					
** **≤ 60	153 (32.9%)	1.00 (ref)			
** **60–70	119 (25.6%)	1.05 (0.75–1.46)	0.76	1.02 (0.61–1.71)	0.94
** **> 70	193 (41.5%)	1.52 (1.14–2.03)	0.004*	1.45 (0.76–2.79)	0.26
**Body mass index (kg/m2)**	27.9 (24.1–31.6)	1.08 (1.06–1.11)	<0.001**	1.01 (0.98–1.04)	0.42
**Comorbidities (n, %)**					
** **Diabetes	264 (56.8%)	1.25 (0.98–1.58)	0.07	1.35 (0.97–1.87)	0.07
** **Chronic Cardiac Disease (Not HTN)	97 (20.9%)	0.93 (0.69–1.25)	0.63	1.10 (0.76–1.60)	0.6
** **Hypertension	259 (55.7%)	1.06 (0.83–1.34)	0.65	0.85 (0.63–1.15)	0.29
** **Chronic Pulmonary Disease	36 (7.7%)	0.85 (0.54–1.33)	0.47	0.78 (0.44–1.38)	0.39
** **Asthma	51 (11%)	0.69 (0.46–1.04)	0.06	0.66 (0.40–1.10)	0.11
** **Chronic Kidney Disease	45 (9.7%)	1.13 (0.77–1.64)	0.52	0.82 (0.50–1.34)	0.42
** **Chronic Liver Disease	5 (1.1%)	1.08 (0.4–2.9)	0.87	1.79 (0.56–5.75)	0.32
** **Neurological Disorder	28 (6%)	0.97 (0.58–1.61)	0.91	0.75 (0.39–1.44)	0.38
** **Malignant Neoplasm	18 (3.9%)	1.33 (0.79–2.4)	0.28	0.79 (0.39–1.44)	0.52
** **Immunosuppression	4 (0.9%)	2.19 (0.82–5.89)	0.12	1.31 (0.42–4.13)	0.64
**Laboratory findings during admission**					
** **Routine blood test					
** **White blood cells, ✕ 109/L	8.1 (5.4–14.1)	1.12 (1.09–1.14)	<0.001**	1.02 (0.98–1.05)	0.26
** **Neutrophils, ✕ 109/L	6.6 (4.8–9.71)	1.07 (1.05–1.1)	<0.001**	1.02 (0.98–1.07)	0.33
** **Lymphocytes, ✕ 109/L	0.7 (0.5–0.9)	0.14 (0.08–0.21)	<0.001**	0.35 (0.18–0.68)	0.002*
** **Platelets, ✕ 109/L	69.3 (112–213)	0.993 (0.991–0.995)	<0.001**	0.998 (0.996–1.00)	0.07
** **Other laboratory values					
** **Procalcitonin (PCT), ng/ml	0.28 (0.07–0.59)	7.83 (5.25–11.65)	<0.001**	1.89 (1.03–3.46)	0.04*
** **High-sensitivity cardiac troponin I, pg/ml	36.9 (5.9–120.4)	1.009 (1.007–1.011)	<0.001**	1.004 (1.002–1.006)	0.001*
** **C-reactive protein (CRP), mg/L	66.1 (39.7–115.6)	1.011 (1.009–1.014)	<0.001**	1.004 (1.001–1.007)	0.005*
** **Ferritin (μg/L)	1072.76 (806.2–2129.3)	1.00 (1.00–1.001)	<0.001**	1.00 (1.00–1.00)	0.47
** **Fibrinogen (mg/dl)	491.4 (383.6–615.1)	1.002 (1.001–1.003)	<0.001**	1.00 (0.99–1.001)	0.57
** **Prothrombin time (s)	14.5 (12.1–16.5)	1.09 (1.04–1.14)	<0.001**	1.04 (0.99–1.10)	0.09
** **D-dimer (ng/ml)	6.35 (1.18–21.33)	1.05 (1.04–1.06)	<0.001**	1.02 (1.001–1.03)	0.04*
**Quick SOFA Score**	1 (1–2)	1.29 (1.12–1.48)	<0.001**	1.02 (0.83–1.25)	0.89
**Quick SOFA Score Grades**					
** **0	46 (9.9%)	1.00 (ref)			
** **1	209 (44.9%)	0.69 (0.44–1.09)	0.12	1.00 (ref)	
** **2	151 (32.5%)	1.26 (0.8–1.97(	0.32	1.07 (0.69–1.64)	
** **3	59 (12.7%)	1.27 (0.76–2.11)	0.35	1.38 (0.94–2.04)	0.77
**Total SOFA score (0–24)**	6 (4–7)	1.12 (1.08–1.26)	<0.001**	1.16 (1.07–1.25)	0.09
**SOFA Risk group interpretation**					< 0.001**
** **≤ 9	427 (91.8%)	1.00 (ref)			
** **10–12	28 (6%)	1.95 (1.32–2.89)	0.001*	0.76 (0.41–1.41)	0.39
** **> 12	10 (2.2%)	1.59 (0.48–3.01)	0.14	0.31 (0.11–0.89)	0.03*
**Type of intubation during admission**					
** **Elective	38 (8.2%)	1.99 (1.33–2.96)	0.001*	1.25 (0.75–2.06)	0.38
** **Emergency	173 (37.2%)	2.63 (2.03–3.41)	<0.001**	1.97 (1.37–2.85)	<0.001**
** **No intubation	254 (54.6%)	1.00 (ref)		1.00 (ref)	
**Developed sepsis / septic shock at during ICU admission**	154 (33.1%)	0.74 (0.57–0.95)	0.018*	0.47 (0.34–0.65)	<0.001**
**Inotropes / vasopressor**	122 (26.2%)	1.72 (1.36–2.19)	<0.001**	0.89 (0.63–1.26)	0.5
**Antibiotic**	433 (93.1%)	0.56 (0.38–0.83)	0.004*	1.19 (0.56–2.54)	0.65
**Major complications events**					
** **Life-threatening hemorrhage	11 (2.4%)	1.39 (0.71–2.71)	0.33	1.35 (0.64–2.87)	0.43
** **Stress myocardiopathy	44 (9.5%)	1.71 (1.203–2.42)	0.003*	2.31 (1.27–4.16)	0.006*
** **Acute kidney injury	127 (27.3%)	1.37 (1.08–1.75)	0.01*	0.70 (0.51–0.96)	0.03*
** **Cardiac arrythmia	88 (18.9%)	1.38 (1.06–1.81)	0.017*	0.82 (0.56–1.19)	0.28
** **Myocarditis	34 (7.3%)	1.23 (0.82–1.83)	0.31	0.78 (0.41–1.49)	0.45
** **Pericardial effusion	29 (6.2%)	1.16 (0.74–1.81)	0.51	1.45 (0.72–2.89)	0.29
** **Pneumothorax	38 (8.2%)	0.7 (0.45–1.07)	0.101	1.01 (0.62–1.66)	0.96
** **Atelectasis	93 (20%)	0.79 (0.60–1.05)	0.14	0.76 (0.54–1.09)	0.14
** **Prolonged delirium	75 (16.1%)	1.13 (0.85–1.51)	0.38	0.85 (0.58–1.24)	0.4
** **Seizure	42 (9%)	1.52 (1.15–1.99)	0.003*	0.75 (0.39–1.39)	0.36

Age > 70 years (HR = 1.52 [1.14, 2.03]), elective intubation (HR = 1.99 [1.33, 2.96]), emergency intubation (HR = 2.63 [2.03, 3.41]), inotrope use (HR = 1.72 [1.36, 2.19]), stress myocardiopathy (HR = 1.71 [1.203, 2.42]), acute kidney injury (HR = 1.37 [1.08, 1.75]), arrhythmia (HR = 1.38 [1.06, 1.81]), and seizure (HR = 1.52 [1.15, 1.99]) were associated with a higher risk of mortality.

Kaplan–Meier survival analysis was performed to assess the association between the study variables and survival. The ICU length of stay was used as the mean time. The distribution of the study variables is summarized in Table 1. A log-rank test was conducted to determine if there were differences in the survival distributions of the study variables. The survival distributions for the following variables were significantly different: higher age groups were associated with lower survival rate (χ^2^(2) = 12.288, p = 0.002), BMI > 30 was associated with lower survival rate (χ^2^(2) = 20.372, p < 0.001) and antibiotic use was significantly associated with better survival (χ^2^(2) = 9.37, p = 0.002).

**Fig 3:**
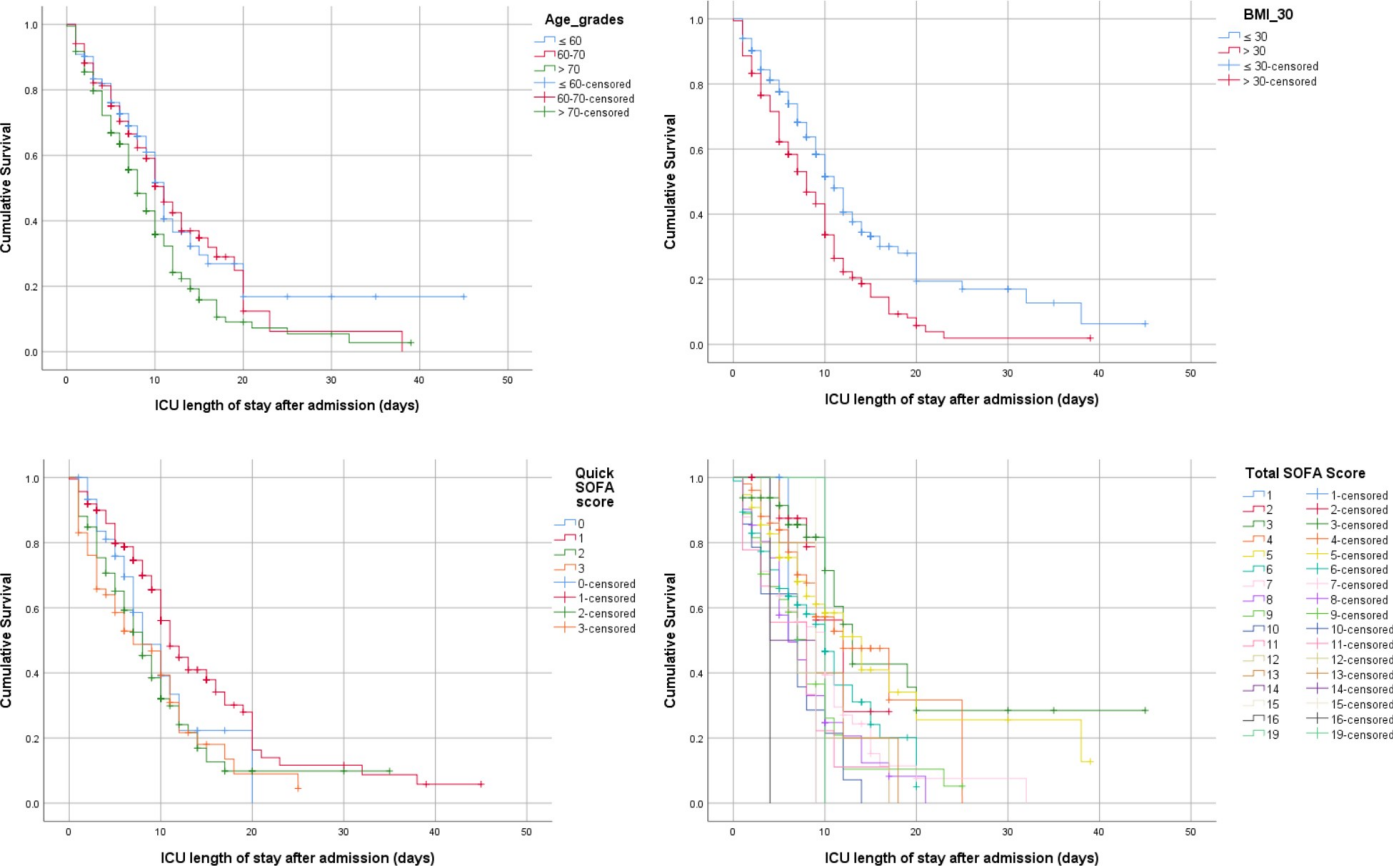
Kaplan–Meier survival estimates during 60 days following ICU admission. (A) Age grades (≤ 60, 60–70, > 70 years). (B) BMI (≤ 30, > 30). (C) Quick SOFA (0, 1, 2, 3). (D) Total SOFA Score.

**Fig 4:**
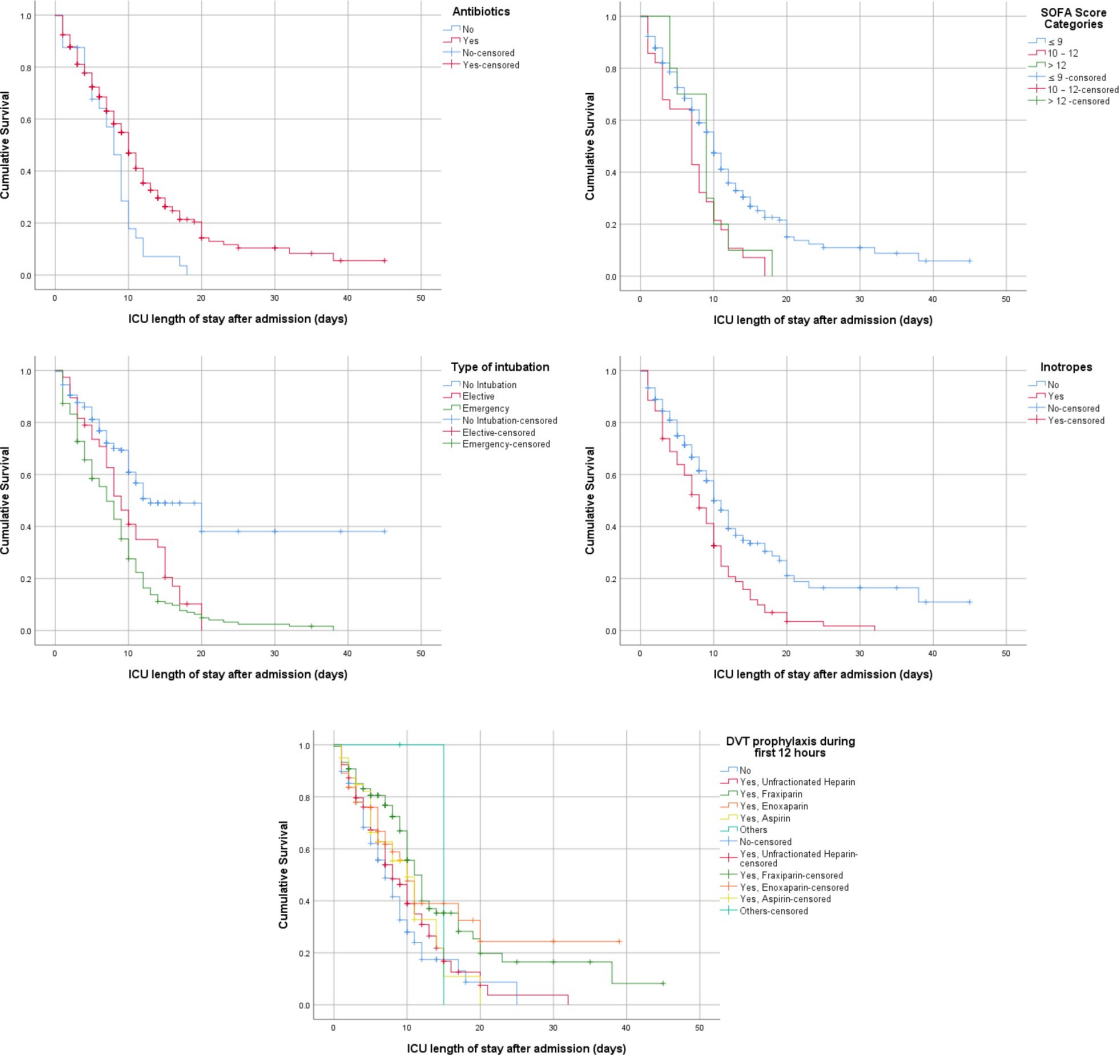
Kaplan–Meier survival estimates during 60 days following ICU admission. (A) Antibiotic (yes, no). (B) SOFA score category (≤ 9, 10–12, >12). (C) Type of intubation (no intubation, elective, emergency). (D) Inotrope use (yes, no). (E) DVT prophylaxis during first 12 hours.

The results found that the quick SOFA and SOFA scores were associated with mortality and survival. Lower quick SOFA scores were associated with better survival (χ^2^(2) = 24.467, p < 0.001). Lower total SOFA scores were significantly associated with better survival outcomes (χ^2^(2) = 50.852, p < 0.001). When dividing the SOFA score into three categories (≤ 9, 10–12, and > 12), the results showed a significant difference in survival based on the SOFA risk score category. High scores were associated with higher mortality (χ^2^(2) = 14.38, p = 0.001), and the inverse was also true. The type of intubation was another variable that could predict mortality; emergency intubation was associated with a high risk of mortality compared to elective or no intubation (χ^2^(2) = 22.45, p = 0.001). Patients who received deep vein thrombosis (DVT) prophylaxis during the first 12 hours of admission had better outcomes and lower mortality (χ^2^(2) = 24.689, p < 0.001). Patients who received inotropes or vasopressors had higher mortality rates than those who did not (χ^2^(2) = 22.45, p < 0.001). When comparing the effects of some newly used treatments, such as convalescent plasma, blood transfusion, remdesivir, tocilizumab, famotidine, and high or low doses of vitamin C or vitamin D; however, the results did not find a significant difference in survival distribution, which may be due to the limited number of patients who received these therapies. The majority of patients received steroids; however, there was no significant difference in the survival distribution (p = 0.43). **Figs 3 and 4** provide Kaplan–Meier estimates of overall survival based on the previous significant variables.

### 3.5 Hospital resources and infrastructure

Regarding hospital facility infrastructure and availability of equipment among the participating hospitals, seven (63.5%) were teaching hospitals, whereas four (36.4%) were not teaching hospitals. The median number of ICU beds (IQR) was 15 (range, 6–16), with a range from 4 to 18 ICU beds. The number of mechanical ventilators was 9 (range, 7–12), with a range from 6 to 20 available devices. The patient-to-nurse ratio ranged from 2 to 12, with a median (IQR) of 4 (range, 2–4). The number of intensivist specialists ranged from 1 to 6 specialists, with a mean (IQR) of 2 (1–4), whereas the number of doctors in training ranged from 2 to 9, with a median (IQR) of 4 (2–6). All hospitals had non-invasive and invasive ventilation facilities. None of the hospitals had extracorporeal membrane oxygenation (ECMO). None of the hospitals had a 24 h visitor policy; only five (45.5%) had a visitor policy of limited hours, and two (18.2%) provided end-of-life visitation. **Table 5** summarizes the hospital infrastructure and facilities.

**Table 5:** Hospital infrastructure and facilities (n = 11).

Variables	N (%) / Median (Q1 –Q3)
**ICU type**	
** **Medical	9 (81.8)
** **Mixed	2 (18.2)
**Number of ICU beds**	15 (6–16)
**Number of mechanical ventilators**	9 (7–12)
**Patient / nurse ratio**	4 (2–4)
**Number of intensivist specialists**	2 (1–4)
**Number of resident doctors in training**	4 (2–6)
**Continues renal replacement therapy (CRRT)**	3 (27.3%)
**Acute peritoneal dialysis**	2 (18.2%)
**Hemodialysis facility**	6 (54.4%)
**Availability of laboratory facility**	
** **Day time	6 (54.4%)
** **24hour	4 (36.4%)
** **Outside the hospital on request	1 (9.1%)
**Availability of intubation team**	
** **24 hours	9 (81.9%)
** **Available upon request	2 (18.2%)
**Available of mouth/hygiene care team**	
** **Day time	4 (36.4%)
** **24 hours	3 (27.3%)
** **None	4 (36.4%)
**Shortage of sedative drugs**	
** **Yes	4 (36.4%)
** **No	7 (63.6%)
**Shortage of analgesic drugs**	
** **Yes	4 (36.4%)
** **No	7 (63.6%)
**Shortage of antibiotic and antimicrobial**	
** **Yes	5 (45.5%)
** **No	6 (54.5%)
**Availability educational or training courses ICU physicians**	
** **Yes	6 (54.5%)
** **No	5 (45.5%)
**Availability of psychological support for healthcare workers**	
** **Yes	1 (9.1%)
** **No	10 (90.9%)
**Accommodation for ICU staff**	
** **Yes	6 (54.5%)
** **No	5 (45.5%)

## 4 Discussion

This study described the clinical characteristics and presentation of critically ill Libyan patients with COVID-19. In the analysis, the study assessed several basic, clinical, laboratory, and management characteristics and outcomes of severely ill patients who were admitted to ICUs, where followed them up to hospital discharge or mortality at 60 days. The main finding is the 60.4% mortality rate following ICU admission, which, to our knowledge, is the highest recorded compared to all countries’ previous studies. The study also found that age, BMI, laboratory findings, admission SOFA score, and quick SOFA score were strong predictive risk factors for mortality.

Mortality among critically ill COVID-19 patients has been reported in several previous studies. However, there is a scarcity of information on COVID-19 status in Africa, which is at a higher risk due to resource limitations and several challenges that are endured by healthcare systems in African countries. One study conducted in several African countries found a mortality rate of 54.7% 30 days after ICU admission [16].

A previous study conducted in several countries revealed different mortality rates and associated risk factors. A study conducted in Wuhan, China, in February 2020, found that among 226 COVID-19 critical care patients, 87 (38.5%) died [25]. Another single-center study in China also found that the mortality rate among 344 critically ill patients was 38.7% (133) at or before 28 days [12]. The study also confirmed that lymphopenia is associated with mortality in critically ill patients with COVID-19. Another single-center study in Spain [13] of 237 critically ill COVID-19 patients found that 55 died and 116 remained in the ICU by April 19, 2020. In a large multicenter prospective study in three European countries including Belgium, France, and Switzerland that included 4224 critically ill patients with COVID-19, the mortality rate was 31% (1298) on day 90 after ICU admission [14]. Another multicenter study in Spain and Andorra [26] of 960 patients reported a mortality rate of 31% (203). Another multicenter prospective study in France [27] of 335 critically ill patients reported a mortality rate of 7.76% (26). In a single-center study in Sweden of 260 participants, mortality was found in 60 of those admitted to the ICU [28]. In Germany, a multicenter prospective study of 223 patients found a mortality rate of 35% (78) in ICU patients [29]. In a large multicenter prospective study in European countries, among 639 patients admitted to ICUs, ICU mortality was 24% [30]. A retrospective multicenter study in Georgia in the United States found a mortality rate of 28.6% (62 out of 217) among critically ill patients with COVID-19 [31]. Another retrospective single center in Ohio [32] of 495 critical care patients found that 91 (18.4%) died in the ICU. A study in Michigan of 141 patients found a mortality frequency of 40.4% (57) at 30 days of admission [33]. In Australia, a study of 18 patients admitted reported death of four (22%) patients in the ICU [34].

Our study demonstrated the utilization of both the SOFA score and quick SOFA score at admission. The results found a median (IQR) quick SOFA score of 1 (1–2) and a total SOFA score of 6 (4–7) on admission. When compared with other studies, a single-center retrospective study in Singapore in 22 patients with a 2/22 mortality rate found an initial admission median (IQR) SOFA score of 2.5 (1.25–7) [35]. A single-center study in Turkey between March and April 2020 found a SOFA score of 4 (3–6) at admission, with a mortality rate of 50.5% (52 out of 103) [36]. Another study of 55 patients in the ICU in the United Arab Emirates reported a SOFA score of 4 (3–7) with a mortality rate of 22% (12) [37]. A prospective multicenter study of 226 COVID-19 critical care patients in China reported a SOFA score of 4 (2–8) at admission [25]. In the COVID-ICU group, a study of 4224 European COVID-19 critical care patients found a SOFA score of 5 (3–8) [14]. In a Spanish study [26], of 960 critically ill patients had a SOFA score of 5 (3–7), and the overall mortality rate was 31% (203 out of 960). In a Swiss single-center prospective study of 129 patients, the SOFA score was 6 (4–7); however, the mortality rate was 19% (24 out of 129) [38]. Another multicenter study conducted in Germany found a SOFA score of 5 (3–9), with a mortality rate of 35% (78 out of 223) [29]. A retrospective study in Canada reported a SOFA score of 5 (3–8), and 18 patients died out of 106 (17%) admitted [39]. Therefore, both SOFA and quick SOFA scores are useful tools for predicting mortality among ICU patients with COVID-19. Our study showed that both were significantly associated with higher ICU mortality. When the quick SOFA score was divided into two categories, 0–1 vs. 2–3. The results showed a significant difference in mortality between the two categories. Similar results were obtained when the total SOFA score was ≤ 9 vs. 10–12 vs. > 12. Both tools indicated that mortality and non-survivorship were significantly associated with higher SOFA or quick SOFA scores. Thus, the quick SOFA score can be used in low-resource settings as an initial assessment of mortality risk to predict outcomes. Calculating the total SOFA score is challenging, especially in low-resource settings, as it requires extensive laboratory results, which can be difficult to obtain in countries with limited resources. Therefore, this recommend the use of quick SOFA as it only requires clinical variables such as Glasgow coma scale < 15, respiratory rate ≥ 22, and systolic blood pressure ≤ 100, in which each one is given one point per answer. Detecting patients with a high risk of mortality can help provide specific care by healthcare providers to reduce the risk of death in the ICU. It can also be a usable tool to prioritize the management of patients at increased risk for complications and mortality, given the treatment and management option limitations.

Our study demonstrated high SOFA and quick SOFA scores at admission, which, when compared to previous reports, indicates the severity of study participants and explains the high mortality observed in this study. Furthermore, this study was conducted during the surge in cases in Libya, when many patients could not be admitted appropriately and in a timely manner to the ICU due to a lack of resources and shortage of ICU beds in comparison to the high number of patients. This resulted in some patients waiting for hours or days to find a suitable ICU bed at COVID-19 isolation centers. This delay in appropriate management carries a high risk of hypoxia and complications, as demonstrated by the high SOFA and quick SOFA scores at admission. The study also observed that some patients had to search for ICU beds in several cities, which has major consequences, especially in frail and elderly patients who cannot tolerate delays in the management of hypoxia and respiratory issues. Another possible explanation is the shortage of healthcare supplies and the lack of adequate training of healthcare workers. Libyan ICUs were not prepared in terms of equipment and facilities to fight COVID-19; there was a severe shortage of supplies and a low number of mechanical ventilators, as demonstrated in a previous report [20]. The lack of training and the low number of trained intensive care specialists is another obstacle, both in Libya and in the rest of Africa. In this study, it reported 2 (1–4) intensive care specialists per hospital and a patient to nurse ratio of 4 (2–4), which are not enough to cover the shifts and manage patients appropriately and accurately.

Therefore, health authorities need to increase the resilience of hospitals, especially during the second wave. Hospitals need to be equipped with more ICU beds to avoid delays in admission and a higher number of mechanical ventilators to treat those patients who present with respiratory compromise. As in our report, the shortage of mechanical ventilators can significantly affect the ability of Libya and other African countries to treat critically ill patients with COVID-19.

In addition, there is a need to recruit more ICU nurses and intensive care specialists to increase the ability of hospitals to manage COVID-19 patients. There is also a need to provide adequate training for healthcare providers and to provide management guidelines that can help utilize the resources and provide hospitals with newly developed drug therapies that have been proven to increase survival and reduce mortality, which are not adequately available in Libya due to their high prices and worldwide demands. However, the government should invest more in healthcare facilities and provide adequate supplies of these drugs in order to mitigate the catastrophic consequences of COVID-19 in critically ill patients, as demonstrated in our report.

Increased age was found to be associated with an increased likelihood of mortality among critically ill patients. The study also found that those aged > 70 years had the highest mortality rate compared to the other age groups, which is in line with previous studies [40]. The results found that increased BMI was associated with a higher risk of mortality among COVID-19 critical care patients. This is in line with previous studies, as obesity and increased BMI carry a risk of higher hospital admission rates, morbidity, and mortality for COVID-19 patients [41, 42].

Increased blood laboratory values, such as white cell count and neutrophils, were significantly associated with higher mortality risk, which is in line with previous reports [43]. In contrast, a lower lymphocyte count was significantly associated with a higher risk of mortality, which has been reported previously [44]. In addition, a lower platelet count was significantly associated with mortality rate, which has also been reported in the literature as a risk factor [45].

Regarding other laboratory findings, procalcitonin levels were significantly associated with a sevenfold higher risk of mortality in critically ill patients with COVID-19, which has also been reported in several previous studies as a predictor of mortality [46]. Other biomarkers, such as cardiac troponin, C-reactive protein, ferritin, fibrinogen, prothrombin time, and D-dimer were shown to be associated with a higher risk of mortality among critically ill COVID-19 patients in our study. These biomarkers have been reported in several studies and meta-analyses regarding their role as risk factors and factors predicting mortality and worsening of COVID-19 severity [47–53].

Antibiotic use was significantly associated with lower mortality, as previously reported. These findings may support the use of antibiotics in COVID-19 patients in order to prevent secondary bacterial infection or infection acquired in the ICU, as patients with sepsis were more likely to die in critical care. However, antibiotic use among COVID-19 patients is still debatable, and some previous reports did not support their use [54].

DVT is considered a potential complication and life-threatening risk factor for COVID-19 patients, as reported previously [55]. This is supported by these finding; which found an increase in the risk of DVT and pulmonary embolism (PE) complications while comparing the frequency between admission and during admission, DVT (0.4% vs. 1.7%), and PE (4.9% vs. 6.9%), and found that 0.6% developed DVT and PE during admission. The number of patients who received low molecular weight heparin (LMWH), such as Fraxiparine and Enoxaparin, as deep venous thrombosis (DVT) prophylaxis during the first 12 hours, was higher in the survivor group than in the non-survivor group, in agreement with a previous report [56].

The incidence of COVID-19 major complications is another major concern. For cardiovascular complications, the study found stress cardiomyopathy in 44 (9.5%) patients, cardiac arrhythmia in 88 (18.9%), and myocarditis in 34 patients (7.3%), all of which were significantly higher in the non-survivor group. Stress cardiomyopathy has been reported to be associated with COVID-19, as its incidence has increased compared to that in the pre-pandemic era (7%–8% vs. 1%–2%) [57]. Cardiac arrhythmia has been reported in the literature as a complication of COVID-19, and a study in Wuhan in China found that 44.4% of critical care patients had arrhythmia [7]. Cardiac arrhythmia may have several underlying mechanisms, including electrophysiological or structural changes caused by viral infection [58]. Myocarditis has also been reported to be associated with COVID-19 infection, although a previous study reported that the majority of patients survived; however, higher proportion of patients with myocarditis in the non-survivor group [59]. Prolonged delirium as a neurocritical issue was observed in 75 (16.1%) of our patients and was significantly associated with the non-survivor group. Delirium as brain dysfunction has been reported to occur at a high rate in a previous international study; up to 54.9% of patients developed some type of delirium [60]. Seizure was observed in 42 (9%) cases and was found to be higher in the non-survivor group than in the survivor group. This was explained in the literature by several hypothesized mechanisms, such as the hypoxic effect of COVID-19 [61] and the ability of SARS-COV-2 to enter the central nervous system through the angiotensin-converting-enzyme-2 (ACE-2) receptor cells or through the olfactory nerve [62–64].

## 5 Limitations

This study has several limitations. First, the study was conducted in a country that has an ongoing civil war and severe financial crisis, which could explain the high mortality rate, which may need to be addressed in other African countries with similar settings. Second, detailed information about ethnicity, race, and other laboratory findings was not provided due to the limited availability of resources, which limited our ability to analyze these factors on mortality. Another limitation is that the study was conducted in a single country, and the results should not be generalized to other parts of the world. Finally, center-to-center changes are possible; however, our study included the main hospitals and healthcare facilities for COVID-19 patients in Libya.

## 6 Conclusion

This is a large multicenter prospective study of COVID-19 patients admitted to the ICU in Libya with detailed laboratory, clinical, and SOFA score characteristics, complications, and mortality up to 60 days following ICU admission as the evaluated outcome. Our study also examined the differences in survival rates and factors contributing to mortality. The study showed one of the highest mortality rates in a country with limited resource settings and ongoing conflict, along with a financial crisis. Several challenges for both the healthcare system and healthcare workers in Libya underlie these results. However, this requires urgent intervention from policymakers and health authorities by financially supporting healthcare centers, providing sufficient mechanical ventilation devices, increasing ICU bed capacity, and increasing the nurse and specialist ratio to patients. In addition, there is an urgent need to support healthcare workers by providing financial compensation, psychological support, and adequate training. Libyan patients with COVID-19 in critical care are at a higher risk of mortality and complications than those in other regions of the world. Several factors were found to be predictive of mortality, which should be considered by clinicians and healthcare policy makers in future planning and support for hospitals and healthcare workers during the ongoing COVID-19 pandemic.

## List of abbreviations

ARDS: Acute respiratory distress syndrome
COVID-19: Coronavirus disease
ICU: Intensive care unit
SARS-CoV-2: Severe acute respiratory syndrome coronavirus 2
